# Manual of Clinical Microscopy and Chemistry

**Published:** 1904-10

**Authors:** 


MANUAL OF CLINICAL MICROSCOPY AND CHEMISTRY.—Prepared for the use of students and practitioners of medicine. By Dr. Hermann Lenhartz, Professor of Medicine and Director of Hospital at Hamburg, etc. Authorized translation from the fourth and last German edition, with notes and additions, by Henry T. Brooks, M. D., Professor of Histology and Pathology at the New York Post-Graduate Medical School and Hospital; member of the New York Academy of Medicine, etc. With 148 illustrations in the text and nine colored plates. Pages 412, octavo. Bound in extra cloth. Price, $3.00 net. F. A. Davis & Company, Publishers, 1914-16 Cherry Street, Philadelphia, Pa.

This is one of the most admirable and timely manuals that have recently appeared. Most doctors are called on to make microscopical examinations of urine or blood or sputa, and often have not time to read up on more elaborate works; hence they will find an epitome of what they want to know very useful and convenient. For students also it will be a great help, in as much as it is elementary to some extent, there being much in it that an expert would find superfluous—the mere a b c, as it were. The colored plates are excellent and will be like an atlas to the student of geography. The work has had a large sale in Germany and all over Europe. Dr. Brooks has evidently supplied a want. D.

